# Dressed to impress: impact of environmental adaptation on the *C* *andida albicans* cell wall

**DOI:** 10.1111/mmi.13020

**Published:** 2015-05-09

**Authors:** Rebecca A. Hall

**Affiliations:** ^1^ School of BiosciencesInstitute of Microbiology and InfectionUniversity of BirminghamEdgbaston Park RoadBirminghamB15 2TTUK

## Abstract

*C* *andida albicans* is an opportunistic fungal pathogen of humans causing superficial mucosal infections and life‐threatening systemic disease. The fungal cell wall is the first point of contact between the invading pathogen and the host innate immune system. As a result, the polysaccharides that comprise the cell wall act as pathogen associated molecular patterns, which govern the host–pathogen interaction. The cell wall is dynamic and responsive to changes in the external environment. Therefore, the host environment plays a critical role in regulating the host–pathogen interaction through modulation of the fungal cell wall. This review focuses on how environmental adaptation modulates the cell wall structure and composition, and the subsequent impact this has on the innate immune recognition of *C* *. albicans*.

## Introduction

*Candida albicans* is a frequent coloniser of the oral, genital and gastrointestinal tracts, and has carriage rates between 30% and 70% in healthy individuals (Perlroth *et al*., 2007). The maintenance of colonisation over invasion is a fine balance between fungal proliferation and innate immune recognition. However, during periods of immune suppression and microbiome dysbiosis, *C. albicans* is able to overcome the immune system and cause disease. In female populations, *C. albicans* causes 75 million cases of genital thrush each year in women of childbearing age, with 5% of these women developing recurring infection (Sobel, 1992; 2007). Oropharyngeal candidiasis is prevalent in HIV/AIDS patients with 90% of infected individuals developing candidiasis during the progression of the HIV infection (Wu *et al*., 2012). In addition to HIV patients, oropharyngeal candidiasis is common (estimated 20–80% of patients) in individuals following radiotherapy to treat head and neck cancers (Davies *et al*., 2006). During periods of trauma, or neutropenia *C. albicans* can disseminate into the bloodstream and cause life‐threatening systemic disease.

During colonisation and disease *C. albicans* is exposed to a variety of environmental signals (pH, temperature, hypoxia, hormones, elevated carbon dioxide partial pressures, nutrient limitation and serum), stresses (oxidative, nitrosative and osmotic stress) and microbial imposed environments (quorum sensing molecules), which drive the expression of virulence factors. The response to these environmental signals is mediate through the cyclic adenosine monophosphate (cAMP)‐dependent Protein Kinase A (PKA) pathway, mitogen‐activated protein kinase cascades (i.e. Hog1, Cek1, and Mkc1) and the pH responsive Rim101 cascade. On the whole, host‐derived environments (elevated CO_2_, serum, 37°C, nutrient and phosphate limitation and alkaline pH) promote the yeast to hyphal switch (Buffo *et al*., 1984; Odds, 1988; Klengel *et al*., 2005; Romanowski *et al*., 2012), whereas microbial‐derived signals (secreted quorum sensing molecules) inhibit morphogenesis, maintaining yeast growth (Hornby *et al*., 2001; Hogan *et al*., 2004; Boon *et al*., 2007; Vílchez *et al*., 2010). In addition to affecting morphology, exposure to environmental signals also affects fungal stress resistance. For instance, quorum‐sensing molecules and alternative carbon sources enhance oxidative stress resistance (Rodaki *et al*., 2009; Deveau *et al*., 2010; Hall *et al*., 2011; Ene *et al*., 2012a).

Despite the wealth of information available on how the local environment impacts on morphogenesis, virulence gene expression and stress resistance, considerably less is known about how the environment regulates the structure and composition of the cell wall. This dynamic external organelle contains many carbohydrate epitopes that are recognised by cells of the innate immune system. Therefore, environmental modulation of the cell wall will regulate the host–pathogen interaction. This review focuses on how adaptation to the host environment affects the cell wall structure and, by drawing parallels from studies on *C. albicans* glycosylation mutants, the consequences these modifications have on the host–pathogen interaction.

## Environments encountered within the host

The environmental parameters encountered within the host depend on the body site the fungus occupies. For example, the oral mucosa is mildly acidic, and the saliva contains mucins, glycoproteins, electrolytes, immunoglogulins and antimicrobial peptides (cathelicidin LL‐37, lactoferrin, histatin 5, α‐defensins and β‐defensins). On the other hand, the mucosa of the female reproductive tract is acidic (pH 4–pH 5.3) and has lactate as the main carbon source, and vaginal secretions contain interferon‐ε, antimicrobial peptides (human beta defensin 2, elafin and MIP3α) and hormones (John *et al*., 2005; Ghosh *et al*., 2010; Ravel *et al*., 2011; Wira *et al*., 2011).

Infection of the mucosa results in the formation of microbial biomasses observed as white plaques. These plaques resemble biofilms and are comprised of *C. albicans* and bacteria from the microbiota (Nett *et al*., 2010). The microbiota of the oral mucosa is comprised of Firmicutes, Bacteriodetes and Protobacteria, with lower numbers of Fusobacteria and Actinobacteria (Segata *et al*., 2012). In contrast, the female reproductive tract is predominately colonised by *Lactobacillus* (Ravel *et al*., 2011; Witkin *et al*., 2013), with smaller numbers of *Staphylococcus*, *Streptococcus*, *Clostridium*, *Escherichia* and *Bifidobacterium* (Ravel *et al*., 2011). Bacteria can communicate with *C. albicans* through several processes. For example, bacteria can interact with *C. albicans* by direct cell–cell interactions (Hogan and Kolter, 2002), although the importance of this interaction on infection outcome is unknown. The most studied fungal–bacterial interaction is the cell density dependent secretion of soluble chemical messengers into the environment known as quorum sensing. The secretion of fungal and bacterial quorum sensing molecules regulates the expression of virulence factors in both the donor and the recipient. *C. albicans* responds to quorum sensing molecules from a variety of bacteria, including *Pseudomonas aeruginosa*, *Burkholderia cenocepacia*, *Streptococcus mutans* and *Enterococcus faecalis* (Hogan *et al*., 2004; Boon *et al*., 2007; Jarosz *et al*., 2009; Cruz *et al*., 2013).

The extracellular matrix that holds the biofilm together restricts the diffusion of quorum sensing molecules and other metabolic byproducts resulting in their accumulation in certain parts of the biofilm. For example, centres of colonies that resemble biofilms have elevated carbon dioxide levels, are fairly hypoxic and have higher concentrations of signalling molecules (Rossignol *et al*., 2009; Hall *et al*., 2010; Cottier *et al*., 2012). As a result, discrete domains form within the biofilm. These subpopulations of cells are exposed to different environmental signals and have specific functions. For example, cells at the periphery are highly metabolically active, whereas cells embedded in the centres of such biomasses are nutrient deprived and quiescent (Davidson *et al*., 2011; Traven *et al*., 2012). Moreover, the growth conditions and environmental signals encountered during biofilm formation regulate the mating switch in *C. albicans* with elevated carbon dioxide levels promoting mating competent phenotypes (Daniels *et al*., 2013). The properties of mating competent and incompetent biofilms are different, with mating incompetent biofilms being impermeable, and fluconazole resistant, while mating competent biofilms can be penetrated by the innate immune system and are not inherently resistant to antifungals (Yi *et al*., 2011; Srikantha *et al*., 2012). Therefore, the environment plays a huge role in orchestrating the properties and composition of fungal biofilms, which are key players in nosocomial infections.

## Environmental regulation of the cell wall

The cell wall of *C. albicans* is the most extensively studied pathogenic fungal cell wall to date. Transmission electron microscopy (TEM) has revealed that the cell wall is a multi‐layered structure (Poulain *et al*., 1978). The inner layer is comprised of chitin and glucans that act as the cell's skeleton, maintaining cell shape and rigidity, while the outer layer is comprised of mannosylated proteins that are essential for cell adhesion, virulence and biofilm formation. These proteins fall into several categories including cell wall associated enzymes (glucanases, proteinases, chitinases), structural proteins, adhesins, receptors/binding proteins, morphology specific proteins (i.e. Hwp1), heat‐shock proteins and glycolytic enzymes (Chaffin *et al*., 1998). These proteins are decorated with simple linear *O*‐linked glycans and complex branched *N*‐linked mannans (Klis, 1994; Klis *et al*., 2001; Bowman and Free, 2006; Free, 2013). These mannans extend away from the fungal cell as fibrils, which can be viewed by high‐pressure freeze substitution TEM (as observed in Netea *et al*., 2006). As these mannans are located on the periphery of the cell wall, they play key roles in innate immune recognition of the fungus.

### Environmental effects on the cell wall proteome

The cell wall is not static, but is a dynamic structure permitting cell expansion, cell division, and morphogenesis. Host and microbial derived environmental signals regulate fungal morphogenesis. Global transcriptional and proteomic studies investigating the effects of these environmental parameters on *C. albicans* identified that many of the cell wall proteins are differentially expressed between different environments (Bensen *et al*., 2004; Hromatka *et al*., 2005; Bruno *et al*., 2010). For example, iron deletion alters the cell wall proteome, resulting in increased expression of cell surface proteins known to function in iron acquisition (Weissman and Kornitzer, 2004). Manipulation of the gaseous environment to levels predicted to occur in the female reproductive tract (restrictive oxygen levels with elevated concentration of carbon dioxide), results in the expression of cell wall modifying enzymes, adhesins, iron assimilation and an enhancement of non‐covalently linked cell wall proteins (Sosinska *et al*., 2008). Growth on alternative carbon sources increases the expression of cell wall biogenesis proteins, adhesion and biofilm related proteins, and environment specific proteins (Ene *et al*., 2012b), confirming that carbon source and metabolism are key mediators in regulating the cell wall structure and composition. The presence or absence of specific proteins within the cell wall will affect its structure and composition, ultimately affecting innate immune recognition. For example, deletion of *RIM101* results in decreased virulence in an oropharyngeal candidiasis infection model (Nobile *et al*., 2008). This effect is not due to defects in morphogenesis, but due to the reduced expression of cell wall proteins and cell wall modifying enzymes (Nobile *et al*., 2008).

Overexpression of glycosylphoshatidylinositol (GPI)‐linked proteins dramatically influences the ability of *C. albicans* to form biofilms, with some mutants displaying an altered cell wall structure. For example, enhanced expression of Pga22, or deletion of Pga22 enhances the formation of mixed *C. albicans* biofilms (Cabral *et al*., 2014). The ultrastructure of both the overexpressing Pga22 and Pga22 deficient cells contain less dense mannan fibril layers (Cabral *et al*., 2014), suggesting that the involvement of Pga22 in biofilm formation is complex. Therefore, environments that promote or repress the expression of Pga22 may aid biofilm formation. As biofilms are a major contributor to nosocomial infections, this discovery may be important for future preventative therapies.

### Environmental impact on cell wall carbohydrates

In addition to affecting the expression of cell wall proteins, growth in different environments affects the carbohydrate content of the cell wall. For example, hyphae have half the amount of mannan, threefold more glucan and up to five times more chitin compared with yeast (Staniszewska *et al*., 2013). Hypoxic environments increase the thickness of the *Aspergillus fumigatus* hyphal cell wall through enhanced β‐glucan levels (Shepardson *et al*., 2013). In addition to increasing biosynthesis, the environment can also influence the structure of these carbohydrates. For example, β‐glucan isolated from *C. albicans* hyphal cells is unique and contains 3,6‐ and 2,3‐linkages in addition to the 1,3‐ and 1,6‐linkages observed in yeast glucan (Lowman *et al*., 2014). Analysis of the structure of the chitin and glucan polymers that are integrated into the cell wall under different environmental conditions is currently limited. However, recent data indicate that the structure and functional characteristics of these polymers may be dependent on the environment. For example, growth in lactate containing media increases the elasticity of the cell wall (Ene *et al*., 2012a). Although the exact mechanism for this observation is unclear, it is likely that alternative cross‐linking of the carbohydrate skeleton of the cell wall plays some role. Analysis of the cross‐linking that occurs in the cell wall under different environmental conditions is warranted to address how such biophysical properties are mediated.

Although chitin only comprises a small amount of the fungal cell wall (3% dry weight), subtle changes in its concentration can have a large impact on fungal biology. Indeed, an increase in chitin levels from 3% to 10% impacts on antifungal sensitivity and recognition by the innate immune system (Mora‐Montes *et al*., 2011). Chitin synthesis is regulated via the Hog1, Pkc1 and calcium signalling pathways (Munro *et al*., 2007). These pathways are central to *C. albicans* biology and are activated by many conditions including osmolality, micronutrient limitation, calcium ions, alkaline pH, thermal tolerance, antifungal drugs, pheromones and oxidative and cationic stress (reviewed in Kraus and Heitman, 2003; Monge *et al*., 2006; Hall *et al*., 2009). Therefore, all these conditions have the potential to modulate the composition of the cell wall.

Chitin can also be deacetylated to chitosan. Chitosan is required for bud separation and to maintain cell wall integrity in vegetative *Cryptococcus neoformans* cells (Baker *et al*., 2007). Studies on chitosan are limited compared with chitin, but it is possible that exposure to different environmental conditions alters the ratio of chitin and chitosan which will have structural and functional consequences on the cell wall.

The structure of *C. albicans N*‐mannan has been largely deduced from nuclear magnetic resonance (NMR) spectroscopy studies. The employment of 1H‐13C NMR to deduce the *N*‐mannan structure was highly successful, permitting the assignment of each linkage in the manno‐oligosaccharide, providing a structural model (Kobayashi *et al*., 1989; 1991; 1994a; 1997; Shibata *et al*., 1989, 1991, 1992a, 1992b, 1995, 2007). More recently, Lowman *et al*. combined 1D and 2D COSY and NOSEY NMR with chemical shift data for the *N*‐mannan side chains to assign resonances to non‐degraded intact *N*‐mannans (Lowman *et al*., 2011). Although this method does not provide detailed structural information, it does permit the rapid identification of changes in mannan composition. Although in its infancy, this methodology has the potential to provide detailed insight into the mannan composition under a variety of conditions.

In terms of changes in mannan content, TEM indicates that the mannan fibrils are longer and more densely packed around the yeast cell body than the extending hyphae (Cheng *et al*., 2011). Hyphal *N*‐mannan contains significantly less phosphomannan and branched α1,6‐manann but increased β1,2‐mannose incorporation into the acid‐stable mannan and higher levels of unsubstituted α1,6‐manann in the α1,6‐backbone (Shibata *et al*., 2007, Fig. 1). The *N*‐mannan of exponentially growing *C. albicans* cells has increased β1,2‐mannose in both the acid‐liable and acid‐stable fractions, which correlates with reduced levels of terminal α1,2‐mannose (Koyama *et al*., 2009). Therefore, even growth phase alters the composition of the cell wall and is an important consideration when elevating immune responses.

**Figure 1:**
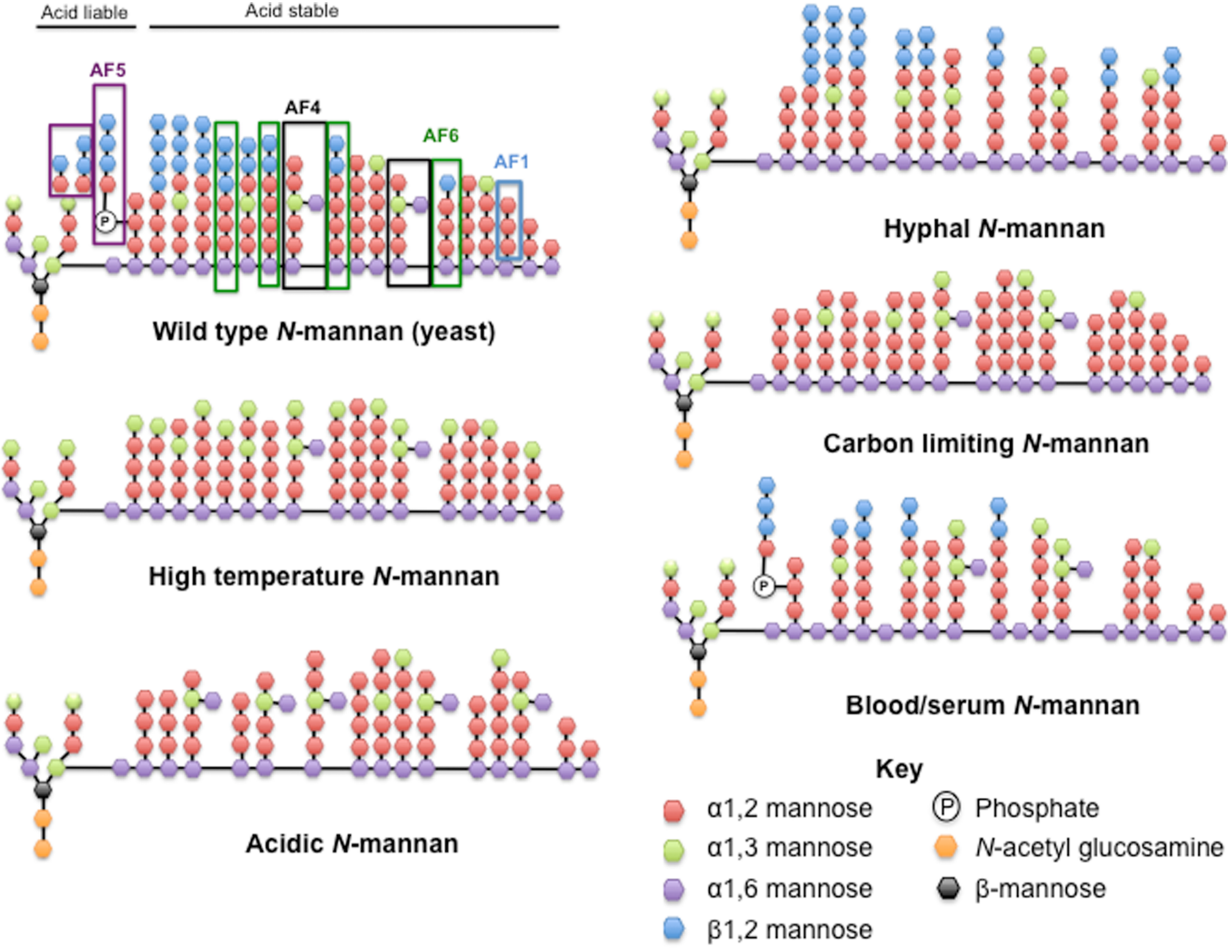
Local environmental conditions influence the *N*‐mannan structure and composition of *C* *. albicans*. Diagrammatic representation of the changes the environment imposes on fungal *N*‐mannan, as determined by a series of NMR studies. Predicted epitopes for *C* *andida* antigen factors mentioned in the text are depicted on the wild‐type mannan.

Within the host, fungi are exposed to elevated concentrations of carbon dioxide, which is important for virulence, morphogenesis and metabolism of *C. albicans* (Klengel *et al*., 2005; Hall *et al*., 2010). However, the impact carbon dioxide levels have on the cell wall have not been investigated. Transmission electron microscopy revealed that elevated carbon dioxide levels enhance the electron density of the outer cell wall layer (Persi *et al*., 1985), suggesting that carbon dioxide regulates mannan content or composition. In agreement with components of fungal metabolism impacting on cell wall composition, the available carbon source also influences the mannan composition, with lactate grown cells displaying less structured fibrils compared with glucose grown cells (Ene *et al*., 2012a). Analysis of the mannan from cells grown in carbon‐limiting conditions, confirms that the β1,2‐mannose content of the cell wall is reduced in both acid‐labile and acid‐stable *N*‐mannan fractions (Tada *et al*., 2008, Fig. 1). Growth on blood or serum containing agar decreases mannan complexity (Lowman *et al*., 2011) and increases mannoprotein content (Kruppa *et al*., 2011). According to Lowman *et al*., the *N*‐mannan extracted from *C. albicans* grown in blood contains reduced terminal β1,2‐mannose units and reduced α1,2‐mannan side chains (Lowman *et al*., 2011). Interestingly, the growth in blood reduces the presence of small side chains in the acid labile fraction but maintains long side chains (Lowman *et al*., 2011, Fig. 1). Therefore, there appears to be selectivity in which acid‐labile side chains remain incorporated in the cell wall.

Growth at elevated temperatures (37–40°C) reduces the agglutination activity of *C. albicans* with antigen factors 4, 5 and 6. However, these cells maintain activity to antigen factor 1, suggesting that cells grown at high temperatures have considerably less β1,2‐mannose but maintain α1,2‐mannose side chains (Okawa and Goto, 2006). Analysis of the phospholipomannan moiety of the cell wall confirms that β1,2‐mannose incorporation remains constant (Trinel *et al*., 2002), suggesting that the reduction in β1,2‐mannose is specific to the *N*‐mannan. Further analysis of the *N*‐mannan structure deduced that the loss of β1,2‐mannose was compensated by an increased incorporation of α1,3‐mannose onto the non‐reducing ends (Okawa and Goto, 2006, Fig. 1). Therefore, temperature has a huge impact on *N*‐mannan composition and is likely to impact on the composition of other cell wall components.

The onset of genital thrush has been linked to microbiome dysbiosis due to antimicrobial treatment. The removal of Lactobacilli from the microbiota results in an increase in the environmental pH promoting morphogenesis of *C. albicans*. Growth in acidic conditions (i.e. below pH 4) leads to cleavage of the phosphodiester bond between the acid stable and acid labile *N*‐mannan (Kobayashi *et al*., 1991) and release of the phosphomannan into the environment. Analysis of the branched *N*‐mannan indicates that acidic pH promotes the inclusion of branched α1,3‐mannose (Kobayashi *et al*., 1994b) and increases terminal α1,3‐mannose into the side chains (Fig. 1). NMR analysis of *N*‐mannan extracted from Serotype A *C. albicans* grown in media buffered at pH 2 confirmed that acidic environments also deplete the *N*‐mannan of β1,2‐mannose units (Kobayashi *et al*., 1994a). Therefore, acidic environments have a profound impact on the *N*‐mannan structure and composition. Other environmental changes associated with vaginitis include fluctuations in oestrogen levels. The occurrence of genital candidiasis positively correlates with oestrogen levels, with oral contraceptives, hormone replacement treatment and pregnancy increasing the prevalence of *Candida* colonisation and vulvovaginal candidiasis (Spinillo *et al*., 1995; Bauters *et al*., 2002). Oestrogen levels have been implicated in regulating fungal morphogenesis and adhesion to the vaginal epithelium (Cheng *et al*., 2006; Kravtsov *et al*., 2014). However, the impact of oestrogen on the fungal cell wall has not been investigated. Gene expression analysis confirms that oestrogen regulates the expression of several cell wall associated proteins (Cheng *et al*., 2006), suggesting that oestrogen is likely to impact on the cell wall structure.

In addition to environmental signals, *C. albicans* encounters several stresses during colonisation of the human host. These include oxidative, osmotic and nitrosative stress, which are utilised as antimicrobial agents by cells of the innate immune system. Osmotic stress induces shortening of the acid‐labile side chains, whereas oxidative stress induces elongation of these side chains (Koyama *et al*., 2009, Fig. 1). These changes in mannan structure are slight, indicating that these stresses do not impact greatly on mannan composition. The affect of nitrosative stress on mannan composition has not been analysed. Considering that nitrosative stress extends the lag phase of fungal growth (Kaloriti *et al*., 2012), and growth phase is important in terms of cell wall composition (Koyama *et al*., 2009), it is likely that nitrosative stress will impact on cell wall biogenesis. In the phagosome *C. albicans* encounters all three stresses simultaneously. To date, the impact of simultaneous stress on the fungal cell wall has not been investigated. Combinatorial environments of oxidative and cationic stress synergise to enhance antifungal activity (Kaloriti *et al*., 2014). Therefore, combinations of environmental stresses are likely to have profound effects on the cell wall composition and ultrastructure.

In addition to environmental stimuli mediating changes in the cell wall, mechanical forces also appear to impact on the cell surface. For example, applying pressure on a single adhesin molecule induces the formation of micro‐domains that span the entire cell wall (Alsteens *et al*., 2010). Although atomic force microscopy is an artificial force, these experiments suggest that adhesin binding under flow (i.e. attachment to blood vessel epithelium) induces conformational changes in the cell wall that aid adhesion and may impact on host innate immune recognition.

## Innate immune recognition of the *C* *andida* cell wall

Environmental parameters within the host regulate the structure of the carbohydrate epitopes displayed on the cell surface. These epitopes act as pathogen‐associated molecular patterns (PAMPs) and are recognised by myeloid pattern recognition receptors (PRRs). For example, β‐glucans are recognised by the phagocytic receptor Dectin‐1 and complement receptor 3 (Thornton *et al*., 1996; Brown and Gordon, 2001), *O*‐mannan through TLR4 (Netea *et al*., 2006), whereas *N*‐mannan is recognised through the Mannose receptor, Dectin‐2, DC‐SIGN, Mincle and Galectin‐3 (Fradin *et al*., 2000; Tada *et al*., 2002; Taylor *et al*., 2004; Rouabhia *et al*., 2005; McGreal *et al*., 2006). Therefore, environments that change the presentation or structure of the PAMPs have the potential to impact on innate immune recognition. In support of this theory, exposing macrophages to different morphological forms of *C. albicans* alters the surface expression of the various PRRs. Yeast cells enhance the expression of TLR4 and Dectin‐1, whereas the expression of TLR2 and Dectin‐2 is increased in the presence of hyphae (Han *et al*., 2013). The differences in the receptor expression profiles are hypothesised to result from the differential exposure and structural reorganisation of cell wall PAMPs. For example, hyphal glucan, which has different linkages to glucan isolated from yeast cells, stimulates a potent immune response activating the inflammasome and inducing processing and secretion of the pro‐inflammatory cytokine IL‐1β, an effect not observed with yeast glucans (Lowman *et al*., 2014). This discrimination between the ability of yeast and hyphal cells to activate the NLRP3 inflammasome has been posed as a mechanism the immune system uses to distinguish commensal (yeast) growth over invasive (hyphal) growth (Cheng *et al*., 2011).

So far, our knowledge regarding the direct implication of growth in different environments on innate immune recognition remains limited. However, glycosylation mutants have highlighted that modification of PAMP expression does impact on immune recognition. For example, deletion of *MNN4*, a gene required for the attachment of the phosphomannan to the *N*‐mannan, results in reduced phosphomannan incorporation into the cell wall (Hobson *et al*., 2004), similar to growth in acidic conditions. The depletion of phosphomannan has been shown to reduce the rate at which *C. albicans* is phagocytosed (McKenzie *et al*., 2010; Sheth *et al*., 2011; Lewis *et al*., 2012), suggesting that phosphomannan is required for efficient phagocytosis. However, studies using purified phosphomannan suggest that the β1,2‐mannose moiety of the phosphomannan inhibits the attachment of *C. albicans* to macrophages (Fradin *et al*., 1996). In agreement with β‐mannose displaying an inhibitory role in innate immune responses, complete removal of β1,2‐mannose enhances proinflammatory cytokine production (Ueno *et al*., 2013), suggesting a role for β‐mannose in masking other proinflammatory epitopes. Therefore, the role of phosphomannan in innate immune recognition is still unclear. As cells grown in acidic environments exhibit similar cell wall traits as the *mnn4*Δ mutant (i.e. loss of phosphomannan), it is plausible that the immune responses would be similar. However, growth in acidic environments will affect more than just the phosphomannan content of the fungal cell wall (i.e. cell wall protein expression). Therefore, the net affect of acidic pH on PAMP exposure and innate immune recognition may be greater than simply removing the phosphomannan.

Growth in lactate, a carbon source encountered during colonisation of the vaginal epithelium, influences cytokine production shifting the immune response from a protective Th17 response to a Th2 response (Ene *et al*., 2013). The alternative PAMP expression observed in lactate grown cells resulted in reduced phagocytosis by the innate immune system (Ene *et al*., 2013). Despite this, lactate‐grown cells escape from macrophages more readily, presumably due to altered elasticity of the cell wall. However, the use of fungal morphogenesis to rupture macrophages and evade the immune system is a controversial topic because it is not observed during *in vivo* infections (Brothers *et al*., 2011). Therefore, the impact of this host–pathogen interaction in fungal disease requires further investigation.

*Candida albicans* mutants defective in *O*‐ and *N*‐mannan biosynthesis also show altered immune responses. Removal of *O*‐mannan from the fungal cell wall significantly reduces IFN‐ϒ, whereas depletion in *N*‐mannan reduced both TNF‐α and IFN‐ϒ secretion from macrophages (Netea *et al*., 2006). Reduction of *O*‐mannan also results in enhanced phagosome maturation in macrophages (Bain *et al*., 2014). Reducing the mannan composition also impacts on the ability of phagocytes to efficiently phagocytose *C. albicans* (McKenzie *et al*., 2010; Sheth *et al*., 2011; Lewis *et al*., 2012). However, *C. albicans* mutants that are almost totally devoid of mannan promote an enhanced pro‐inflammatory response, due to the exposure of the underlying β‐glucan (Hall *et al*., 2013).

Studies using glycosylation mutants must be interpreted with care because deletion in key cell wall biosynthetic genes has an impact on the whole cell wall structure, not just the specific epitope. For example, deletion of Och1 is commonly used for its depleted mannan levels. However, the cell wall of the *och1*Δ mutant contains elevated glucan and chitin levels compared with wild‐type cells and also displays an altered cell wall proteome (Bates *et al*., 2006). So far, an in‐depth analysis of the glucan and chitin structures within the cell wall of the *och1*Δ mutant has not been performed, and our knowledge of the proteome is limited. Therefore, attributing alterations in the innate immune response to the loss of mannan in this mutant needs careful consideration.

Extrapolation of results between *Candida* species also requires consideration. A recent study investigating the role of mannan biosynthesis genes in *C. glabrata* identified that deletion of key enzymes involved in mannan biosynthesis results in hypervirulence (West *et al*., 2013). This is in contradiction with studies on *C. albicans*, where deletion of mannan biosynthetic genes results in reduced virulence (Hall and Gow, 2013). Therefore, environmental perturbation in the fungal cell wall, and the consequence these modifications have on the immune response are likely to be species specific.

Other studies investigating murine vasculitis have shed some light on the importance of growth conditions for host–pathogen interactions. Vasculitis can be induced in mice through the injection of cell wall extracts. Tada *et al*. showed that the degree of coronary arteritis induced in this model is dependent on environment in which the fungal cells were grown. Cell wall extracts from cells grown in carbon‐limiting media at 27°C and 37°C induced significant arteritis, while only cell wall extracts from cells grown in YPD at 37°C induced arteritis (Tada *et al*., 2008). These results also correlated with the ability of the cell wall extracts to induce acute anaphylactoid shock (Tada *et al*., 2008). The major differences in the mannan composition between cells grow in YPD and carbon‐limiting media is the incorporation of β1,2‐mannose into the acid‐stable and acid‐labile *N*‐mannan (Tada *et al*., 2008), suggesting that β1,2‐mannose may inhibit cell wall induced arteritis and anaphylactoid shock.

Recently, Mora‐Montes *et al*. showed that fungal cells possessing more chitin in the cell wall elicit a weaker pro‐inflammatory response than wild‐type cells (Mora‐Montes *et al*., 2011). Interestingly, treatment with the echinocandin class of antifungals promotes chitin synthesis to compensate for the reduced glucan content in the cell wall (Lee *et al*., 2012). Therefore, during treatment, *C. albicans* is exposed to exogenous antifungals that impact on its cell wall structure and composition, and ultimately affect the ability of the host immune system to recognise the invading pathogen. In agreement with this, elevated chitin levels reduce Dectin‐1 dependency *in vivo*, despite cells displaying β‐glucan on the cell surface (Marakalala *et al*., 2013). Although the exact mechanism behind the reduced Dectin‐1 dependency is unknown, it is clear that adaptation of the fungal cell within the host plays a major role in regulating the host–pathogen interaction.

Polymicrobial interactions also play a major role in regulating the innate immune response. For example, coinfection with *C. albicans* and *Staphylococcus aureus* in peritonitis enhances the proinflammatory response, significantly increasing mortality (Peters and Noverr, 2013). Bacteria are able to bind fungal hyphae (Hogan and Kolter, 2002; Peleg *et al*., 2008; Silverman *et al*., 2010; Peters *et al*., 2012), but the direct consequence of this binding on disease progression is unknown. One hypothesis is that binding enhances bacterial dissemination (Schlecht *et al*., 2015). However, in *C. albicans* and *S. aureus* coinfections dissemination is dependent on cohabitation, but not fungal morphology (Nash *et al*., 2014). Furthermore, binding of *P. aeruginosa* to *C. albicans* hyphae is antagonistic, rather than agonistic (Hogan and Kolter, 2002), suggesting that a more complex interaction occurs in polymicrobial infections. Components of the cell wall including adhesion proteins and carbohydrates are important mediators of bacterial attachment (Brand *et al*., 2008; Ovchinnikova *et al*., 2013; Dutton *et al*., 2014), often requiring several components (extensively reviewed in Demuyser *et al*., 2014). Considering the observation that bacteria preferentially colonise hyphae, and the cell wall composition varies greatly dependent on morphology, the role of the environment in governing polymicrobial interactions during infection should be addressed. In addition, the impact of bacterial adhesion on the fungus and the fungal cell wall composition are unknown. Therefore, the role of polymicrobial interactions in shaping immune responses through modulation of the fungal cell wall requires investigation.

## Summary

During growth within the host, fungi are exposed to a wide range of environmental conditions, which have a pronounced impact on fungal morphology, stress resistance and virulence. However, we are only just beginning to understand how these niche specific environments modulate the structure and composition of the fungal cell wall, a dynamic organelle require for innate immune recognition. Advances in the emerging field will link together our understanding of environmental sensing with our knowledge of innate immune recognition of fungi to reveal a broad picture of the specific host–pathogen interactions that occur during infection. These new insights into niche specific host–pathogen interactions may identify novel diagnostic markers, antifungal drug targets for future exploration, and highlight the fungal cell wall as a key sensor of the host environment.
